# A Scalable Double‐Sided Wet Etching Strategy for Large‐Area Mushroom‐Shaped Bionic Adhesives

**DOI:** 10.1002/advs.77709

**Published:** 2026-09-11

**Authors:** Keju Ji, Xinran Xu, Jian Chen, Wenjie Chen, Xiaolei Zhu, Haixiang Ma, Xingyu Jiang, Changhong Linghu, Zhendong Dai

**Affiliations:** ^1^ Jiangsu Key Laboratory of Bionic Materials and Equipment College of Mechanical and Electrical Engineering Nanjing University of Aeronautics and Astronautics Nanjing China; ^2^ Department of Mechanical Engineering College of Engineering City University of Hong Kong Hong Kong China

**Keywords:** bionic adhesive materials, double‐sided wet etching, large‐area fabrication, mushroom‐shaped microstructures, scalable manufacturing

## Abstract

Mushroom‐shaped tip architectures play a central role in enhancing the performance of bionic adhesives by amplifying adhesion strength through optimized stress distribution and increased interfacial contact. However, current approaches for fabricating molds of complex adhesive structures are often constrained by high cost, limited repeatability, and limited ability to fabricate complex 3D geometries. These challenges highlight a critical need for high‐strength 3D metal molds that support the scalable manufacturing of mushroom‐shaped adhesive architectures. Here, this work introduces a scalable manufacturing route using coordinated double‐sided non‐simultaneous wet etching. This approach enables precise morphological control and large‑area fabrication of mushroom‑shaped microstructures in a one‐step subtractive process within tens of minutes. By regulating the etching time and mask dimensions, key geometrical parameters, including the necking position and taper angle, can be accurately and reproducibly tuned. The replicated Adhesive structures exhibit high adhesion strength of up to 17.37  N cm^−2^ under ambient conditions, its performance retention in a vacuum environment reaches 98.26 %, alongside excellent surface adaptability, reproducibility, and resistance to micro‐vibrations. Overall, this work provides an efficient strategy for manufacturing high‐performance, 3D bionic adhesives.

## Graphical abstract

This work presents a scalable manufacturing strategy for high‐performance, large‐area, mushroom‐shaped bio‐inspired adhesives. Using a double‐sided wet etching process, the complex 3D metal molds are fabricated rapidly and controllably. The resulting adhesive structures exhibit strong, stable adhesion under various conditions, including atmosphere and vacuum, offering a practical solution for applications requiring stable and consistent attachment.

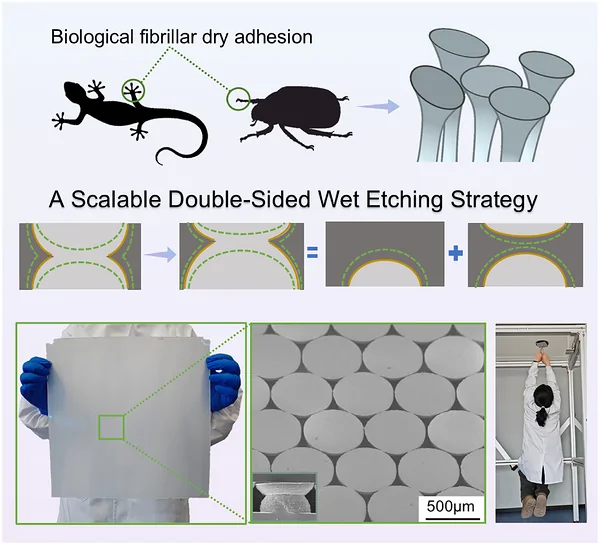

## 1 Introduction

The hairy attachment systems found in various animal groups, such as beetles and geckos, feature surfaces covered with fine patterns of protuberances. These biological structures give rise to fibrillar dry adhesion. mushroom‐shaped tip architectures inspired by beetles have been widely adopted to the performance of bionic dry adhesives [1, 2, 3]. Owing to their characteristic expanded‐tip geometry, these structures can redistribute interfacial stresses, particularly near the contact edge, thereby hindering crack initiation and propagation during detachment. In addition, the compliant tip geometry enables effective adaptation to surface roughness across multiple length scales. The resulting dry adhesion is largely based on interfacial van der Waals interactions enabled by micro‐ and nanoscale physical contacts [4, 5, 6]. Compared with conventional adhesion strategies, such as chemical bonding [7, 8], negative pressure suction [9, 10, 11], and electromagnetic adsorption [12, 13], mushroom‐shaped bionic dry adhesives offer superior compliance, reversibility, and environmental robustness, particularly under conditions involving temperature variation, vacuum operation, and diverse adherend materials and surface states. As a result, mushroom‐shaped adhesive architectures have enabled impactful advances in fields including soft robotics [14, 15], flexible and wearable electronics [16, 17, 18], and reversible transfer and manipulation technologies [19, 20].

However, the manufacturing of high‐performance mushroom‐shaped 3D structures remains challenging, particularly at the large‐area scale. Except for limited cases based on in situ growth [21, 22], most practical fabrication routes rely on transfer or replica molding, in which the achievable structural fidelity, yield, and throughput are fundamentally dictated by the quality and reliability of the negative molds. Consequently, the lack of robust and scalable mold fabrication strategies has become a critical bottleneck in the large‐area manufacturing of mushroom‐shaped adhesive structures.

Existing approaches for mold fabrication can be broadly classified into two categories. The first involves direct fabrication of negative molds using micro‐ and nano‐fabrication techniques, such as micro‐nano 3D printing [23, 24, 25], photolithography [26, 27, 28], and two‐photon polymerization [29].

Among these, two‐photon polymerization [30, 31] enables complex 3D geometries with exceptional resolution; however, its serial, point‐by‐point writing process inherently results in low throughput, severely limiting its applicability for large‐area production. Silicon‐based molds produced via deep etching can generate high‐aspect‐ratio features but suffer from intrinsic brittleness, making them prone to fracture during demolding and repeated replication. Hirai et al. observed that microfabricated silicon molds seldom survive beyond 20 damage‐free demolding cycles [32]. Photolithography, while mature and scalable, is fundamentally a 2D pattern‐transfer technique; although nontrivial surface morphologies can be achieved through approaches such as double‐sided exposure and thermal reflow [33, 34], the reliable fabrication of undercut mushroom‐like profiles with high fidelity and scalability remains challenging.

The second category relies on secondary transfer or reshaping based on these primary molds [35]. Soft molds can partially alleviate the high cost and fragility associated with silicon‐based molds; however, high‐aspect‐ratio microstructures are susceptible to deformation or collapse during demolding and repeated use, particularly under the combined effects of adhesion and capillary forces. Kim et al. observed that the surface roughness of the silicone mold changed within 20 cycles and the mold failed at around 40 cycles [36]. Although metallization strategies, such as vacuum deposition or electroplating, can convert soft masters into metal molds with improved mechanical durability [37], these approaches are generally limited in processing efficiency and achievable geometric complexity. For example, in fabricating a nickel mold with annular grooves via combined electroplating and dry etching, Petersen et al. required 12 h for the electroplating step alone [38].

Although metal molds are intrinsically durable, direct metal processing techniques, including laser machining and precision cutting, are largely confined to 2D features (e.g., pillar‐through holes or wedge‐like structures), making them unsuitable for producing complex 3D mushroom‐shaped profiles [39].

A key challenge for these bio‐inspired structures is their significant performance loss on rough or contaminated surfaces. The intimate molecular contact required for adhesion is compromised on rough topography due to reduced real contact area, and by interfacial fouling from dust or oil [40, 41]. Consequently, future advancements must address not only manufacturing bottlenecks but also the need for adaptive, customized microstructures that can maintain performance on complex, real‐world surfaces.

In this context, wet etching provides a promising route for forming complex 3D metallic morphologies via controlled chemical corrosion, with inherent advantages in process simplicity and large‐area compatibility [42]. Although various micro‐ and nano‐manufacturing techniques can produce intricate structures, their industrial scalability remains limited by high mold attrition, stringent process control, challenges in handling heterogeneous geometries, and high cost. Consequently, developing controllable wet‐etching strategies for directly realizing complex 3D mushroom‐shaped metallic architectures represents a timely and compelling direction for scalable mold manufacturing.

**FIGURE 1:**
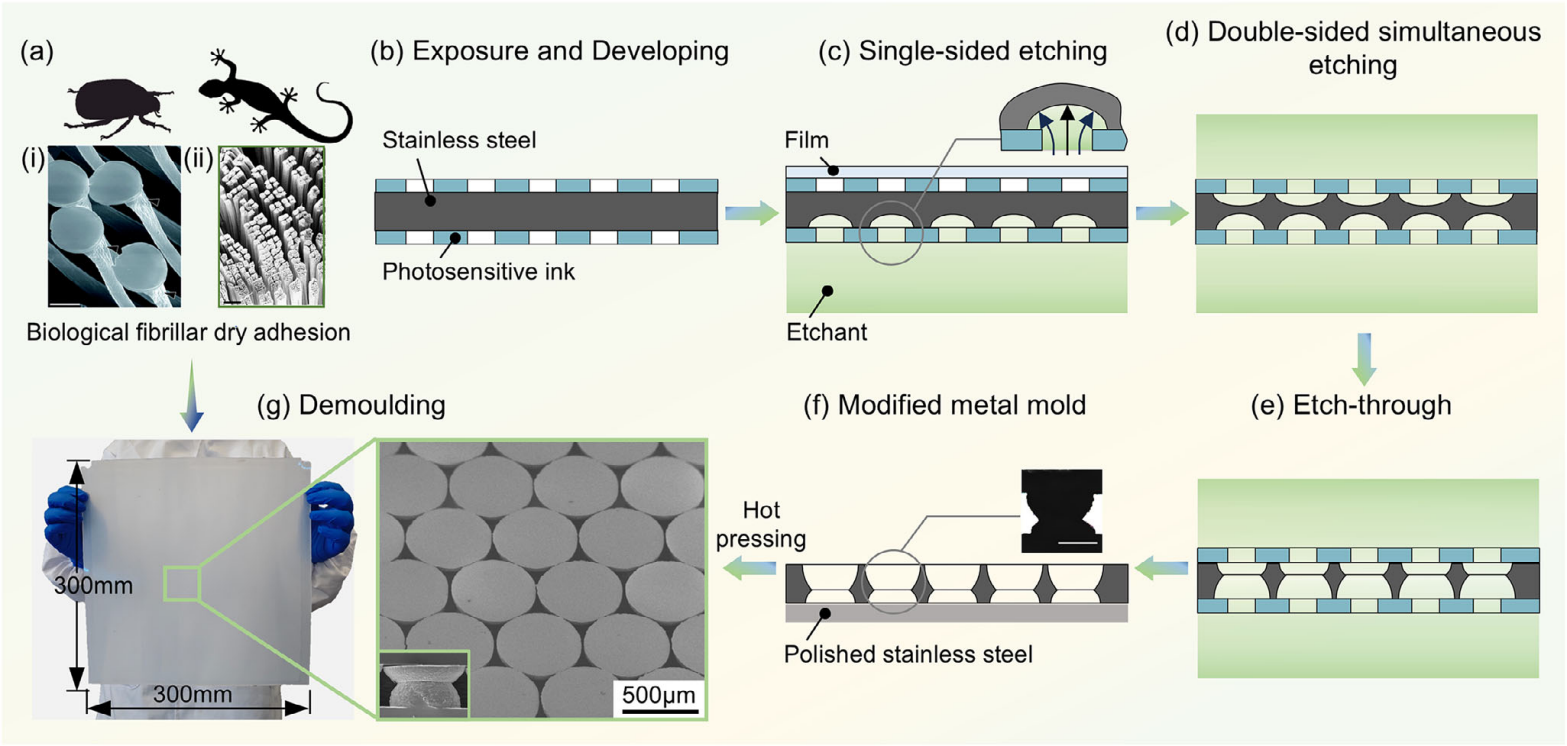
Scalable Manufacturing of bionic adhesive materials with mushroom‐shaped tips using metal molds prepared using a double‐sided wet etching strategy. (a) Schematic of biological fibrillar dry adhesion. i) Mushroom‐shaped adhesive tips of beetle (Reproduced from [2] with permission. Copyright 2018, The Royal Society of Chemistry, Scale bar is 5 µm). ii) Structure of the gecko adhesive setae. Scale bar is 20 µm. (b‐g) Illustrations showing the scalable manufacturing technique of large‐area mushroom‐shaped adhesive structures via double‐sided wet etching: b) Exposure and developing. (c‐e) Double‐sided non‐simultaneous etching. (f) Modified metal mold. (g) Photograph of the fabricated large‐area bionic adhesive materials (30 × 30cm^2^) and the corresponding SEM images of the microstructures and a cross‐sectional view of a single unit.

This study presents a scalable manufacturing strategy for large‐area bionic adhesive materials based on metal molds fabricated via coordinated double‐sided, non‐simultaneous wet etching. By integrating wet etching with efficient platen vulcanization pressing, this approach enables the production of adhesive structures featuring well‐defined mushroom‐shaped microstructures. Precise morphological control is achieved through non‐simultaneous etching combined with tunable photomask designs (Figure 1). Investigation of the underlying process principles and shape‐regulation mechanisms further enables rational structural optimization for enhanced adhesion. The resulting adhesive microstructures exhibit strong adhesion at room temperature, minimal performance attenuation under vacuum, stable adhesion under vibration, and excellent cyclic stability, as well as consistent and reliable adhesion across diverse substrate geometries and material types. Collectively, these attributes establish a scalable and robust manufacturing route that supports industrial‐scale production and practical deployment of bionic adhesive materials across a broad range of application scenarios.

## 2 Results and Discussion

### 2.1 Manufacturing of Metal Molds

The metal mold plays a critical role in determining the geometry and fidelity of the resulting adhesive microstructures. In this study, 304 stainless‐steel sheets were used as the mold substrate, with dimensions of 100 mm × 100 mm × 0.3 mm and surface roughness (Ra) of 0.245 µm. Prior to processing, the stainless‐steel sheets were cleaned with a 10 wt.% oxalic acid solution to remove surface contaminants such as oils, oxides, and other impurities, thereby ensuring stable adhesion of the photoresist layer.

The complete etching process is schematically illustrated in Figure 1. The photoresist was stored in a lightproof environment before use and subsequently applied to both sides of the stainless‐steel substrate by roller coating, yielding a uniform thickness of approximately 40 µm. The coated substrates were prebaked at 120°C for 20 min to evaporate residual solvents and enhance adhesion between the photoresist and the metal surface. After cooling, the substrates were sandwiched between aligned photomasks (Figure S1) and exposed to ultraviolet (UV) light at an exposure energy of 200 mJ cm^−2^ for 10 s. Development was carried out by immersing the exposed substrates in a 10 wt.% Na_2_CO_3_ aqueous solution at 30–40°C for 30 s to selectively remove the exposed photoresist, as shown in Figure 1b.

Subsequently, the patterned substrates were etched using a double‐sided etching machine (JZSK6‐20GD2, Precision Etching Technology Co., Ltd.) at a conveyor speed of 0.15 m min^−1^ (as shown in Figure 1c‐e) using a non‐simultaneous etching strategy. During etching, the etchant nozzles oscillated perpendicularly to the conveying direction with a spray pressure of 0.08 MPa to ensure uniform etchant coverage on both sides of the substrate. The etching parameters (including time and mask size) were carefully controlled to achieve the desired 3D through‐hole structures. After etching, the remaining photoresist was completely removed by immersing the substrates in a 5 wt.% NaOH solution at 50–80°C for 5 min, yielding metal molds with well‐defined and tunable 3D structural features.

### 2.2 Manufacturing of Adhesives With Mushroom‐Shaped Microstructures

The adhesive structure was fabricated using a vacuum‐assisted platen vulcanization and hot pressing process (Figure 1f,g) [43]. High‐tensile‐strength solid silicone rubber was first cut into small pieces and weighed, followed by compounding with a vulcanizing agent at a weight ratio of 100:1. The mixture was homogenized using a two‐roll mill.

To facilitate demolding, a release treatment was applied to the mold cavities to lower the surface energy prior to pressing process. The compounded mixture was subsequently transferred into the prepared metal mold which placed in a platen vulcanizer. Under heated vacuum environment, a high‑temperature‑resistant silicone film positioned at the bottom of the mold conforms intimately to the mold surface, thereby preventing resin leakage. The assembly was subsequently hot‑pressed at 20 MPa and 175°C for 5 min. Owing to the solid silicone rubber's high fracture elongation (571.39%) and low Young's modulus (8.17 MPa), along with the low surface energy of the treated mold, the cured adhesive structure could be cleanly released from the metal mold after cooling (Figure S2). Through this process, a bionic adhesive material with the predefined microstructures was obtained (Figure 1g), achieving a final thickness of approximately 500 µm.

### 2.3 Morphological Control of Metal Molds With Mushroom‐Shaped Cavities

Wet etching transfers predefined patterns onto metal surfaces through controlled chemical reactions, and the resulting morphology is governed by several key parameters, including etching time, etchant concentration, and temperature. In this study, a ferric‐ion‐based (Fe^3^^+^) etchant was employed to micromachine the stainless steel. To isolate the effects of geometric and temporal factors, the etching process was conducted with the etchant concentration fixed at 40 Bé and the temperature maintained at 40°C throughout (Figure S3).

**FIGURE 2:**
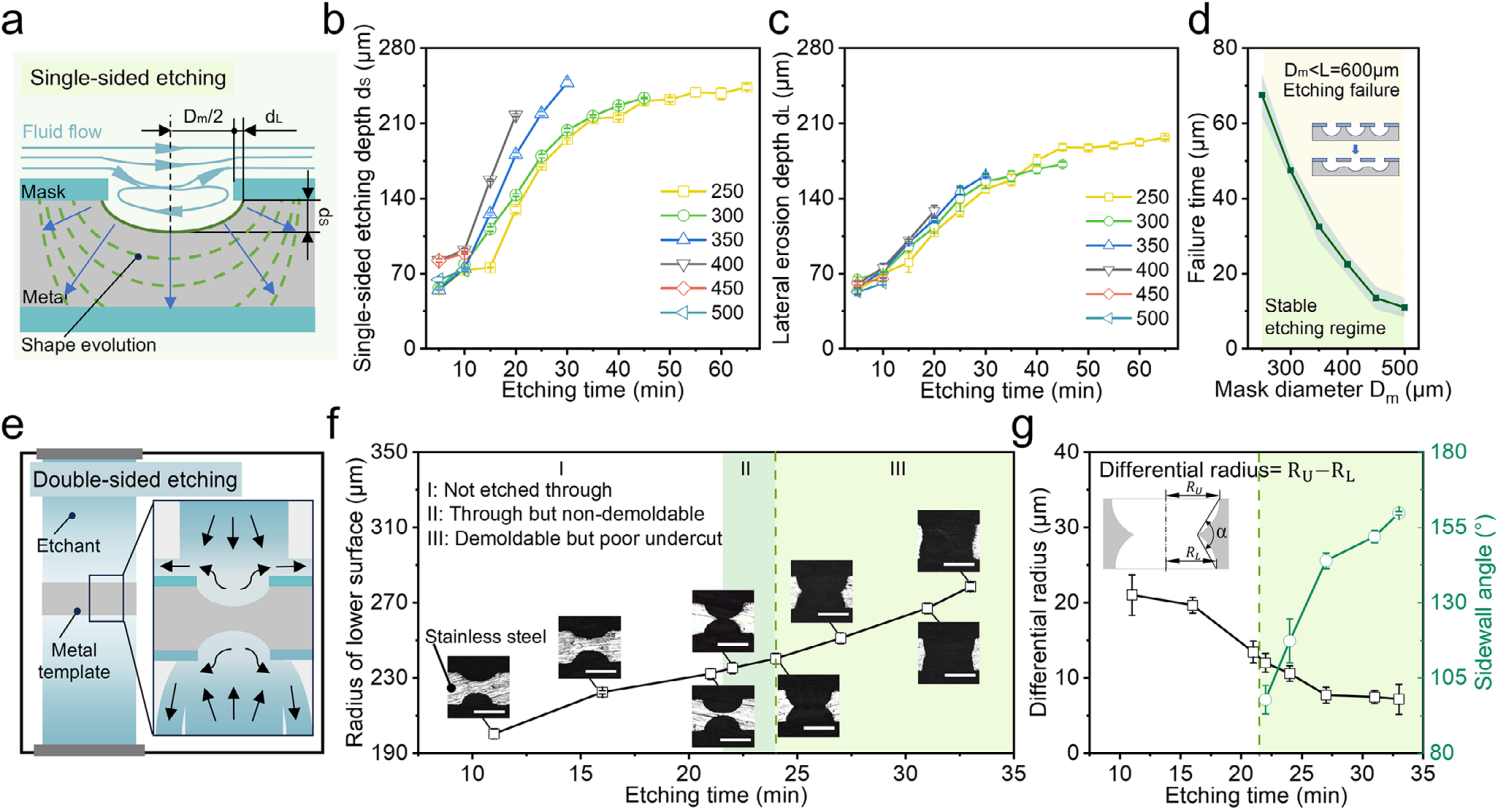
Process Optimization of Etching for Controllable Microstructure in the Stainless‐Steel Metal Mold. (a) Morphology evolution of microstructures during single‐sided etching. (b) Etch depth and Lateral erosion depth as functions of etching time for different mask sizes. (c) Lateral erosion depth versus etching time for different photomask sizes. (d) The dependence of etching failure time on mask size. (e) Schematic illustrating the flow field during double‐sided simultaneous etching. (f) The lower radius for different etching time in the double‐sided simultaneous etching process and cross‐sections of the mold for the corresponding times. (g) Changes in the radius difference between upper/lower surfaces and the sidewall taper angle with double‐sided simultaneous etching time.

During the first‐step single‐sided etching (Figure 1c), the etchant transport is dominated by forced convection regulated by the nozzle pressure. The etchant is atomized by the nozzle and sprayed onto the substrate, forming a thin, continuous liquid film. Continuous droplet impingement generates strong shear flow along the substrate surface. Importantly, the instantaneous morphology of the etching cavity strongly affects the distribution of the local etch rate along its walls, which in turn governs the subsequent cavity evolution (Figure 2a).

To elucidate the relationship between etching time and microstructure evolution, single‐sided etching was performed on patterned metal substrates (2 × 2 cm^2^) with different mask sizes ranging from 250 to 600 µm at intervals of 50 µm. The etching time was varied in 5‐min increments, starting at 0 min and continuing until structural failure was observed (Figure 2d). The etch depth was measured using a laser confocal microscope (LEXT OSL5000, Olympus, Japan). For a fixed mask size, the etch rate exhibited a non‐monotonic trend, initially increasing and then decreasing with etching time. The temporal evolution of surface roughness at the cavity bottom for a fixed mask size of 250 µm parallels the trend in etching rate (Figure S4). In subsequent material preparation, surface roughness enhances demolding resistance via mechanical interlocking; nevertheless, it did not impede the release of the solid silicone rubber.

The etching rate is governed by both reactant mass transfer to the cavity bottom and surface chemical reaction rate. During the early stage of etching, efficient mass transport via forced convection in the shallow cavity allows for rapid replenishment of fresh etchant and removal of products, and maintains a high reactant concentration. Microscopic defects on the metal surface serve as preferential active sites, and the ensuing selective etching leads to the formation of etch pits, protrusions, which sharply increases the surface roughness (Ra). Under these conditions, the etch rate is primarily controlled by the surface chemical reaction, as evidenced by a linear relationship between etch depth and time. However, as etching proceeds, cavity deepening alters the local flow field, leading to the formation of recirculation vortices that hinder material exchange. The resulting accumulation of reaction products and depletion of reactants at the cavity bottom shifts the rate‐limiting step from surface reaction to diffusion control, thereby reducing the etch rate [44, 45]. At this stage, the protrusions are etched away more rapidly, whereas the recessed regions evolve slowly. This decreases the surface roughness (Ra) gradually. Consequently, the mass transfer coefficient, which dictates the reactant diffusion rate, depends on flow conditions, cavity geometry, and reactant diffusivity.

Figure 2b,c further demonstrate the correlation between the photomask size (*D_m_*) and the resulting depth in two distinct directions. Larger mask sizes (e.g., *D_m_* = 500 µm) enhance material exchange, yielding faster initial etching rates, while smaller size experiences stronger diffusion limitations (Figure 2b). After the metal layer is etched through, the initial through‐hole forms, which exhibit an inverted conical morphology (wider at the top and narrower at the bottom). Subsequently, preferential etching at the bottom of the through‐hole by high‐concentration etchant enlarges its diameter and yields a more vertical sidewall [46].

Prolonged etching induced lateral undercut regions of adjacent cavities to coalesce of adjacent etch cavities (Figure S5). It leads to delamination of the photoresist layer from the substrate and the onset of uncontrolled global etching, which substantially reduces the effective substrate thickness. This failure mechanism originates from the coupling of lateral undercutting in adjacent areas. Etch‐through is defined by the merging of undercut regions beneath the mask This occurs once the lateral etch depth exceeds a critical threshold determined by the mask spacing, thereby severely compromising photoresist adhesion stability.

Conversely, a sufficiently large ratio of mask spacing to plate thickness (L/T) prevents this coalescence by enabling independent cavity evolution. To quantify the failure etching time for a given mask size, the time (*T*_0_) at which the data curves stopped in Figure 2b was first recorded. The exact failure time was then pinpointed through replicate experiments after observing failure within the window from *T*_0_ to (*T*_0_ + 5) min. As shown in Figure 2d, the failure time is inversely proportional to mask size. Given a fixed mask spacing of 600 µm, the maximum allowable lateral etch depth at a mask size of 550 µm is about 40 µm (Figure S6). It is less than lateral erosion depth that occurs within the initial first 5 min. To prevent failure, the mask size was capped at 500 µm, adjusted in 50 µm increments.

During double‐sided simultaneous etching, a vortex stagnation zone was formed by liquid buildup on the upper surface. This limits the diffusion of reaction byproducts (Fe^2^^+^) and consequently suppresses the local etch rate. In contrast, spray flow on the lower surface is augmented by gravity and inertial forces. Droplets spread rapidly and are efficiently removed upon impingement, promoting continuous renewal of the liquid etchant film, and leading to a higher etch rate (Figure 2e). Consequently, through‐holes etched simultaneously from both sides exhibit pronounced asymmetry.

To quantify this effect, simultaneous double‐sided etching was performed using identical photomasks with a diameter of 250 µm on both sides of the metal substrate. Etching times ranged from 11 to 33 min, with non‑uniform intervals selected to effectively capture the rapid morphological evolution. Throughout the process, the radius on the lower surface (*R_L_*) was consistently 3.6–12.5% smaller than that on the upper surface radius (*R_U_*) (Figure 2g). This intrinsic asymmetry dictates that the target surface must face upward during etching to form the correct mushroom‐shaped tips.

The etching process of the metal mold progresses through four distinct stages with increasing etching time (Figure 2f). Stage I involves incomplete etching, where two cavities from double‐sided etching remain independent. Stage II leads to the formation of a through‐hole; however, the neck diameter is excessively small, resulting in mold release accompanied by structural tearing. In Stage III, full penetration is achieved, but the sidewall taper angle becomes excessively large, compromising the geometric advantages of the mushroom‐shaped architecture. As shown in Figure 2g, once through‐etching occurs, the sidewall taper angle (α) increases significantly from 97.76 ± 4.75° to 159.69 ± 0.56°, while the neck diameter *D_M_* approaches the upper opening diameter *D_U_*. This trend indicates that etching time must be precisely controlled to control the necking ratio and avoid over‐etching.

**FIGURE 3:**
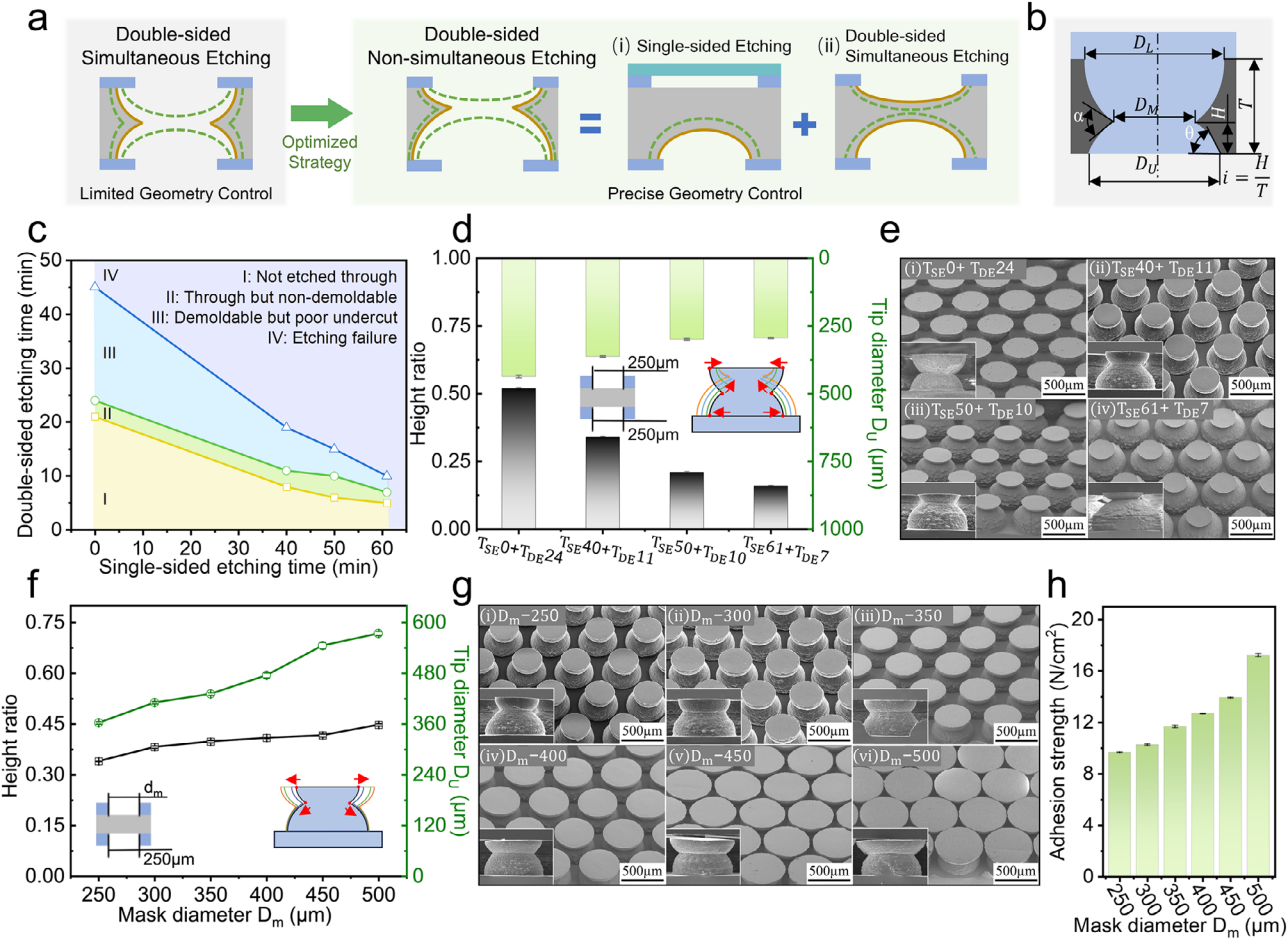
Morphological Tuning of bionic adhesive structure with mushroom‐shaped tips. (a) Schematic of the optimized fabrication process. (b) Schematic of the dimensions of a single bionic adhesive structure. (c) Effect of etching time on the metal mold etching status. (d) Influence of etching time on the geometric characteristics of the mushroom‐shaped cavities in the metal mold. (e) SEM images of microstructures fabricated with different etching time. (f) Effect of mask size on the dimensions of the fabricated mushroom‐shaped adhesive structures. (g) SEM images of adhesive microstructures fabricated with metal molds prepared using different mask sizes. D_m_‐250 and T_SE_ 40 + T_DE_ 11 are the same sample with mask size of 250 µm and Single‐sided etching time of 40 min, double‐sided etching time of 11 min. (h) Adhesive performance of adhesive samples prepared with different mask sizes.

To overcome the limited controllability of tip height (typically constrained to a ratio of ∼0.5) in the standard double‐sided simultaneous etching process, a sequential non‐simultaneous etching strategy was developed. This approach creates a time differential by first performing single‐sided etching on the lower surface while protecting the upper surface with a chemical‐resistant film, resulting in a hemispherical cavity. Subsequently, after removing the protective film, synchronous double‐sided etching is initiated. This sequence increases the depth difference between the two sides (Figure 3a). The process continues until the residual metal layer reaches a critical thickness, the cavities from both sides connect to form a through‐hole with a minimal neck diameter. The vortex field accelerates central dissolution, precisely forming the characteristic necking structure that is the core feature of the mushroom‐shaped microstructure. The dimensions and position of this neck can be accurately controlled by adjusting the initial mask size and the etching time for each step.

### 2.4 Morphological Tuning of Adhesive Structures With Mushroom‐Shaped Tips

The characteristic dimensions of the fabricated microstructure include the bottom diameter of the microstructure (*D*_L_), the end diameter (*D*_U_), and the smallest middle diameter (*D*_M_). The total height of microstructures equals the metal plate thickness *T* (300 µm), while the height of the mushroom‐shaped tip is defined as *H*. The ratio *i* = *H* / *T*, represents the ratio of the expanded height to the total height. *D*_L_ / *D*_M_ is the necking ratio. 𝜃 is the taper angle of the expanded tip. α is the sidewall taper angle of the through‐hole neck. The center‐to‐center spacing between adjacent structures is 600 µm (Figure 3b).

To investigate morphological control via etching time, experiments employed a fixed mask size (*D*_m_ = 250 µm). Single‐sided etching time (*T*_SE_) and double‐sided etching time (*T*_DE_) were varied across a 65 min range, with non‑uniform sampling intervals, to effectively capture rapid shape evolution. When *T*_SE_ was fixed at 40 min, varying *T*_DE_ still produced four distinct morphological stages. The definitions of the first three stages remained unchanged from the earlier description. Stage IV represents etching failure, where adjacent cavities merge. The double‐sided etching time primarily affects sidewall perpendicularity and thus enabling precise control over the taper angle and necking ratio (Figure 3c).

The key to fabricating a high‐quality mold for the adhesive structure with mushroom‐shaped tips lies in achieving a structure that allows for mold release while minimizing the sidewall taper angle. The etching time condition that satisfies these requirements defines the boundary between Stage II (etched through but not releasable) and Stage III (etched through but with excessive taper angle). Accordingly, for a process starting with *T*_SE_ = 0 min, *T*_DE_ of 24 min was selected (Figure 2e). Conversely, with fixed *T*_DE_, varying *T*_SE_ could cause incomplete etching or over‐etching. Therefore, samples prepared at the boundary conditions were selected (Figure 3e). The height ratio *i* decreases significantly from 0.52 ± 0.003 to 0.18 ± 0.002 as *T*_SE_ increases from 0 to 60 min. To prevent structural failure from over‐etching, *T*_DE_ was reduced from 24 to 7 min. It decreases the end diameter from 436.09 ± 3.84 µm to 294.40 ± 2.33 µm and reduces the contact area per unit material by approximately 70% (Figure 3d). Representative SEM images of these microstructures (denoted as *T*_SE_ a + *T*_DE_ b) are shown in Figure 3e.

To further investigate the effect of mask size (*D*_m_) on the final morphology of the fabricated adhesive structure, a specific etching schedule (*T*_SE_: 40 min; *T*_DE_: 11 min) was selected for further experiments. This schedule ensures the total etching time remains below the limiting failure time for all mask sizes, while maximizing the lower surface etch depth and maintaining a relatively large necking ratio for *D_m_*‐500. Under these conditions, increasing *D_m_* from 300 to 500 µm leads to the expansion of the tip diameter of microstructure from 362.64 ± 3.03 µm to 574.10 ± 5.01 µm, and the increase of the height ratio *i* from 0.34 ± 0.002 to 0.44 ± 0.003 (Figure 3f). This increase is attributed to the greater etch depth achievable with a larger mask opening at the same time.

These results confirm that mask size is a primary regulator of the interfacial contact area, enabling a 78% enhancement in the actual contact area per unit material with limited impact on the neck position. Furthermore, mask size significantly influences the interfacial adhesion performance of the structure (Figure 3h), as will be detailed in the discussion. Corresponding microstructures are shown in Figure 3g, where *D_m_*_‐_c denotes a mask diameter of c µm.

The morphological control stems from the synergistic effect of three key parameters. First, the single‐sided etching time primarily determines the depth difference, directly setting the height ratio *i*. Second, the double‐sided etching time, particularly after through‐hole formation, mainly controls the taper angle and thus the necking ratio. Third, the mask size, while influencing etch depth, predominantly dictates the final tip diameter and contact area. The allowable range for both etching times is constrained by the failure time, which itself depends on the mask size. The three parameters act synergistically to enable cross‐scale morphological tuning of the microstructures.

### 2.5 Adhesive Properties Comparison With Flat Pillars in the Atmosphere

The morphology of the end‐expanded structures can be modulated by adjusting the etching process parameters. Normal adhesion force tests were performed on six adhesive structures samples with different geometries (*D_m_*‐250, *D_m_*‐300, *D_m_*‐350, *D_m_*‐400, *D_m_*‐450, *D_m_*‐500). Details of the adhesion tests are provided in Section 4 (Experimental Section). The significant variability in their adhesive performance was presented. According to previous studies [47], the adhesion force (*F*) is proportional to contact area *A*:

(1) $$F∼\sqrt{G_{c}}\sqrt{A/C}$$

where *G_c_* is the critical strain energy release rate, which is set by the interfacial materials, *C* is a constant related to the elastic modulus (*E*), *A* is the total actual contact area between the bionic adhesive materials and the substrate. Thus, a larger contact area induces a larger adhesion force [48].

By adjusting the photomask dimensions, the diameter of the structure's upper surface was substantially expanded, leading to a notable increase in the actual contact area per nominal unit area. Consequently, the resulting adhesion strength increases with the diameter of the upper surface of the mushroom‐shaped adhesive structures, with *D_m_*‐500 owning the maxima under the same loading conditions (see details in Figure S7). The *D_m_*‐500 (Figure 3h) has an adhesion strength of 17.37 N cm^−2^ under a retraction speed of 0.5 mm s^−1^, which increases to 20.10 N cm^−2^ at an elevated retraction speed of 1.5 mm s^−1^ (see details in Figure S7).

**FIGURE 4:**
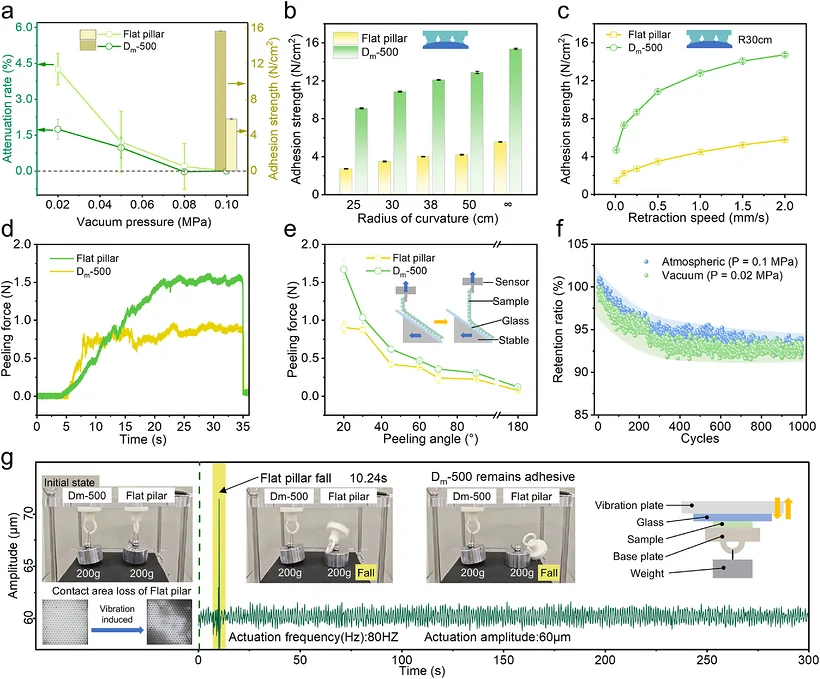
Adhesion performance characterization of the fabricated mushroom‐shaped adhesive microstructures. (a) Adhesion strength on flat surfaces under vacuum for the flat pillar and *D_m_*‐500. (b) Adhesion strength on convex surfaces with different radius of curvature under 0.02 MPa vacuum for the flat pillar and *D_m_*‐500. (c) Adhesion strength as a function of the retraction speed on a convex surface (30 cm radius, 0.02 MPa vacuum pressure). (d) Peeling force‐time curves for the flat pillar and *D_m_*‐500 at a peel angle of 20°. (e) Peeling force as a function of peeling angle for the flat pillar and *D_m_*‐500. (f) Cyclic adhesion performance of *D_m_*‐500 over 1000 cycles at atmospheric pressure and 0.02 MPa vacuum. (g) Adhesion performance under a vibration environment for the flat pillar and *D_m_*‐500.

To investigate the effect of structural features on adhesion strength, flat pillars were also fabricated as a control. The diameter of the flat pillar is the same as the end diameter of *D_m_*‐500. As shown by the columns in Figure 4a, the flat pillars exhibited a maximum adhesion strength of 5.7 N cm^−2^ under a retraction speed of 0.5 mm s^−1^, which is significantly lower (less than 0.5 folds) than that of the *D_m_*‐500 (Figure S8). The enhanced adhesion observed in the *D_m_*‐500 mushroom‐shaped adhesive structure is attributed to the mushroom‐shaped tips, which effectively suppress stress concentration at the contact interface.

### 2.6 Comprehensive Performance Evaluation Under Challenging Conditions

The comprehensive adhesive performance of the fabricated adhesive structures was further evaluated under challenging conditions, including low‐pressure, curved substrate, inclined peeling, repeated loading, and vibrational environments, using custom‐built testing systems (see details in section 4).

As shown in Figure 4a, the adhesion strength exhibits a nonlinear dependence on vacuum pressure under reduced‐pressure conditions. As the pressure decreases from atmospheric pressure (0.1 MPa) to low vacuum (0.02 MPa), both the flat pillar structure and the *D_m_*‐500 mushroom‐shaped structure experience attenuation in adhesion strength, albeit to markedly different extents. Specifically, the flat pillar shows a pronounced reduction of 4.26%, which is approximately 2.5 times greater than that observed for the *D_m_*‐500 structure (1.74%).

Previous studies have reported adhesion attenuation of the adhesive structure under vacuum conditions of around 10–30% [49, 50, 51, 52], which is commonly attributed to the diminished contribution of pressure‐dependent suction effects. However, the adhesion reduction of the fabricated mushroom‐shaped adhesive structures, is obviously much smaller.

To elucidate the origin of the exceptionally low attenuation observed for the *D_m_*‐500 structure, comparative analyses were performed between *D_m_*‐500 configurations with high taper angles and structures with small taper angles, while maintaining identical nominal contact areas. Finite element simulations based on a cohesive zone model were conducted to examine the contact formation and detachment processes between these structures and a rigid flat surface (see details in the Supporting Information). The key model parameters are summarized in Figure S9, and the resulting detachment dynamics, adhesion force, and work of adhesion were systematically analyzed.

The simulation results reveal that the adhesion forces and corresponding adhesion work are comparable for expanded tips with different taper angles. However, structures with a low taper angle promote cavity formation at the contact edge during detachment due to their thinner peripheral geometry (see details in Figure S10). This cavity formation enhances the contribution of suction‐related forces, which become sensitive to ambient pressure and thus lead to greater adhesion attenuation under vacuum conditions [53]. In contrast, the high‐taper‐angle mushroom‐shaped tips effectively suppress cavity nucleation during separation, rendering the suction contribution negligible. As a result, the adhesion of the *D_m_*‐500 structure remains predominantly governed by solid–solid interfacial interactions, leading to a significantly reduced sensitivity to vacuum pressure.

The influence of substrate curvature on adhesion was investigated under a vacuum of 0.02 MPa (Figure 4b). As the radius of curvature decreases from infinity (flat) to 25 cm (convex), the adhesion strength of all microstructures declines (Figure S11), primarily due to a reduction in the actual contact area. Notably, *D_m_*‐500 consistently maintains a clear performance advantage across the entire curvature range. For a convex substrate with a 30 cm radius of curvature, the adhesion strength of *D_m_*‐500 sustains as high as 10.7 N cm^−2^, despite an attenuation exceeding 30% compared to a flat surface. The value is 40% higher than that of the flat pillar structure and is comparable to that of a gecko. The foot of a Tokay gecko (Gekko gecko) can produce 10 N of adhesive force with approximately 100 mm^2^ of pad area [5, 54]. This extraordinary performance of adhesive samples stems from the mushroom‐shaped tip's ability to suppress edge stress concentration, thereby enhancing adaptability to curved surfaces.

At a fixed radius of curvature (30 cm) and a vacuum pressure of 0.02 MPa, the adhesion strength of two microstructures increases rapidly with increasing retraction speed from 0.01 to 2 mm s^−1^. Specifically, the adhesion strength for *D_m_*‐500 increases from 4.70 to 14.73 N cm^−2^ (Figure 4c), indicating a pronounced rate‐dependent adhesion response.

The peeling behavior of the adhesive structures was evaluated as shown in Figure 4d,e. The peeling force remains nearly constant during peeling, indicative of stable interfacial detachment. The adhesive structure with mushroom‐shaped tips (*D_m_*‐500) consistently sustains a higher peeling force than the flat pillar structure. At a peeling angle of 20°, *D_m_*‐500 exhibits a peeling force of 1.56 ± 0.07 N, which decreases monotonically with increasing peeling angle. This trend reflects the reduction in the effective normal adhesive component as the peel angle increases. These results demonstrate that *D_m_*‐500 combines strong adhesion with facile and controllable peeling behavior.

The cyclic durability of *D_m_*‐500 was further examined over 1000 attachment–detachment cycles. A gradual adhesion decay of 7.3% is observed during the first 400 cycles, followed by stabilization of the adhesion strength. This initial decrease is attributed to irreversible plastic deformation and microstructural rearrangement of the adhesive tips, which promotes more intimate interfacial contact while reducing the effective contact perimeter. Beyond 400 cycles, the adhesion strength stabilizes at 92.5 ± 0.3% of its initial value, with fluctuations below 3.2% between 400 and 1000 cycles (Figure 4f). A similar durability trend is observed under vacuum condition (0.002 MPa), confirming excellent long‐term stability.

Vibration resistance of the mushroom‐shaped tip structure (*D_m_*‐500) was evaluated under a normal load of 200 g load using a shaker operating at a frequency of 80 Hz and an amplitude of 60 µm, with the flat adhesive pillar as a control. The flat pillar detaches first at only 10.24 s, whereas the *D_m_*‐500 sample maintains secure adhesion throughout the entire test duration (Figure 4g), highlighting their superior resistance to dynamic perturbations.

In practical robotic and mechanical systems, vibration is unavoidable due to actuator motion, environmental disturbances, and structural resonance. The demonstrated vibration resistance of the mushroom‐shaped adhesive structure therefore represents a critical functional advantage, enhancing energy dissipation and enabling stable attachment under dynamic conditions without additional mechanical fixation or active control, and substantially enhancing reliability in real‐world applications.

**FIGURE 5:**
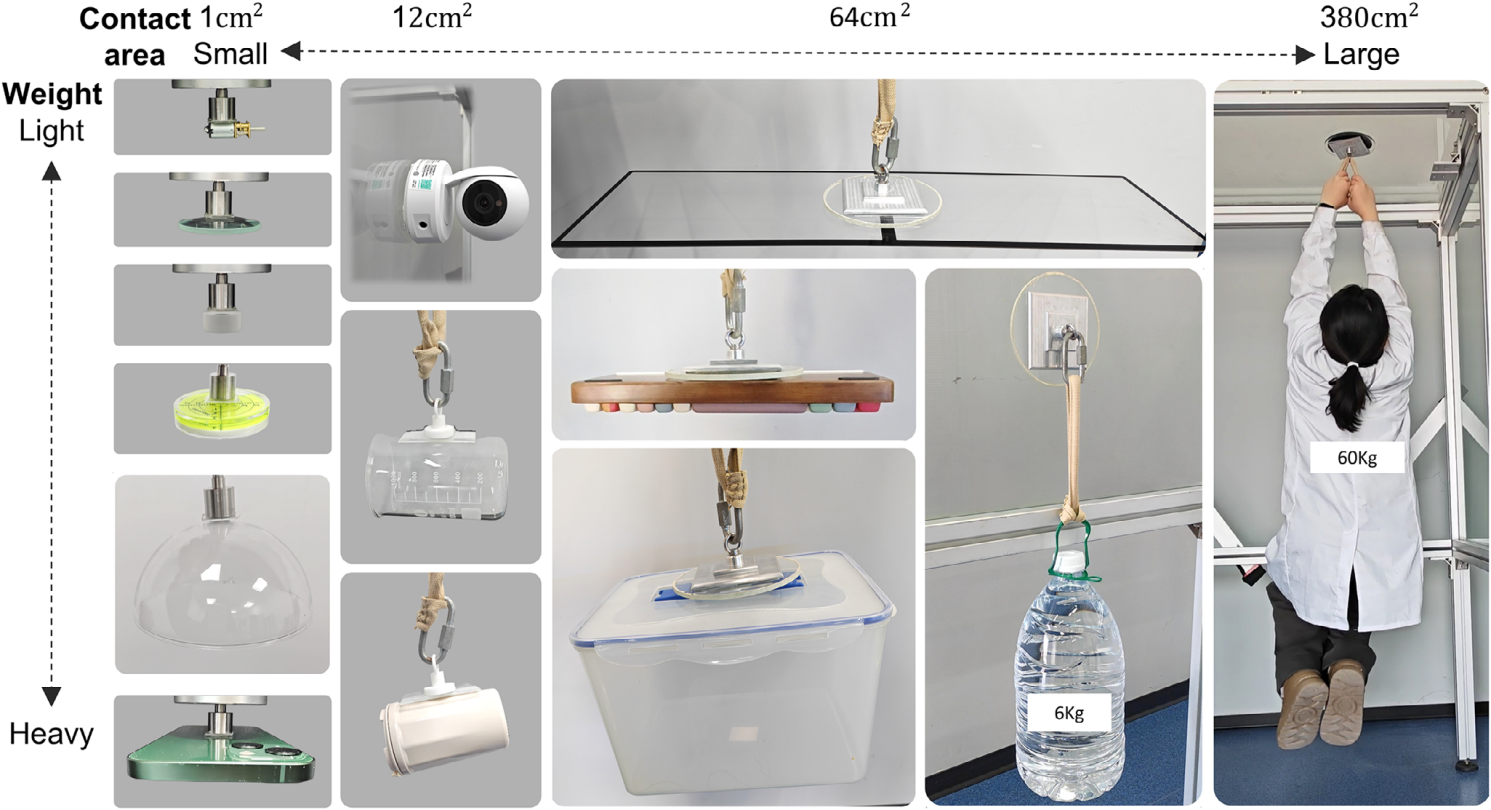
Demonstration of the size scalability and load‐carrying capability of the mushroom‐shaped adhesive structure on diverse target surfaces. The adhesive reliably handles objects spanning a wide mass range, from lightweight (10 g) to heavy loads (up to 6 kg under shear and 60 kg under normal loading). Stable adhesion is achieved across substrates with varying curvatures, ranging from highly curved surfaces (radius of curvature ≈ 8 cm) to flat surfaces, and across diverse material interfaces including metal, plastic, glass, and wood.

Beyond laboratory‐scale testing, the mushroom‐shaped adhesive structures demonstrate strong practical applicability enabled by the scalability of the proposed manufacturing strategy. Figure 5 shows adhesive samples fabricated at different sizes, with contact areas spanning from the centimeter scale to several hundred square centimeters, highlighting the capability of the same fabrication route to produce adhesive structures across a broad size range. Correspondingly, the system sustains shear loads of up to 6 kg and normal loads of up to 60 kg (comparable to the weight of an adult human), enabling reliable handling of objects ranging from lightweight items (10 g) to heavy loads (60 kg). Stable adhesion is maintained across substrates with radius of curvature ranging from highly curved surfaces (≈8 cm) to flat geometries, as well as across diverse material interfaces including metal, plastic, glass, and wood. Collectively, these results demonstrate that the proposed scalable fabrication approach enables large‐area manufacturing of mushroom‐shaped adhesive structures with precisely controlled geometry. Comparative analysis with the prior work [22, 33, 35, 36, 39, 43, 55, 56] further establishes that the material retains high adhesion strength while offering outstanding vacuum stability and durability (Table S1). These properties result in robust adhesive systems well suited for real‐world manipulation and attachment scenarios, showing significant potential for industrial application.

## 3 Conclusions

In this work, a scalable manufacturing strategy was developed for large‐area bionic adhesive materials by integrating coordinated double‐sided, non‐simultaneous wet etching with platen vulcanization pressing. This approach enables the fabrication of high‐strength 3D metal molds with precisely controllable mushroom‐shaped microstructures, overcoming the intrinsic geometric and process limitations of conventional single‐sided and simultaneous double‐sided etching. By elucidating the hydrodynamic and mass‐transfer mechanisms during wet etching, the introduction of a deliberate time offset between the two sides was identified as the key to achieving accurate control over neck position, taper angle, and overall microstructural geometry.

The resulting mushroom‐shaped adhesive structures exhibit strong, robust, and environmentally tolerant adhesion. The optimized architecture shows minimal adhesion attenuation under reduced pressure (only 1.74% as the ambient pressure decreases from 0.1 to 0.02 MPa), good adaptability to curved substrates, stable adhesion under dynamic loading and vibration, and outstanding durability over repeated attachment–detachment cycles (retaining over 92% of the initial adhesion after 1000 cycles). These performance advantages originate from the expanded‐tip geometry, which suppresses interfacial stress concentration, limits cavity‐induced suction effects, and enhances energy dissipation under dynamic perturbations.

Importantly, the proposed fabrication strategy enables size‐scalable production of mushroom‐shaped adhesive structures using the same manufacturing route, supporting reliable adhesion from lightweight objects (10 g) to heavy loads (up to 6 kg in shear and 60 kg in normal loading), across a wide range of surface curvatures and material interfaces. Together, these attributes enable a practical adhesive system that maintains reliable performance across varying loads, surface geometries, and operating conditions.

Overall, this work provides an industrially compatible route for the large‐area manufacturing of high‐performance bionic adhesive materials. Beyond mushroom‐shaped adhesives, the wet‐etching‐based mold design strategy offers general design principles for constructing complex 3D metallic microstructures, with broad implications for scalable microstructure fabrication in soft robotics, wearable devices, and advanced interfacial engineering.

However, this work primarily validates a controllable fabrication route for complex metal molds. The current process still faces inherent limitations when targeting smaller feature sizes, higher aspect ratios, or overhang structures, due to factors such as mask resolution constraints and the isotropic nature of the wet etching mechanism, among others.

To overcome 3D and morphological limits and achieve predictive process design, future work could focus on: employing higher‐precision masks, developing anisotropic etching strategies, and implementing active flow‐field control. More importantly, establishing a multi‐scale computational model that couples mass transfer, reaction kinetics, and geometric evolution would quantitatively reveal the morphological evolution. This would enable the prediction of complex 3D mushroom‐shaped topographies, thereby shifting the process from empirical optimization to mechanism‐guided design. Ultimately, this will lay the foundation for developing next‐generation bionic adhesives with higher adhesive force density, superior directional control, and smarter interfacial functionality, representing a promising direction for further exploration.

## 4 Experimental Section

### 4.1 Materials

The tensile stress‐strain curves of the tear‐resistant silicone (S6001, China) were obtained by testing using a universal tensile machine (JZL‐D, JINGZHUO TESTING INSTRUMENT) (Figure S12). Its Young's modulus of 8.17 MPa and elongation at break of 571.39% can withstand the pull‐out force of the material during demolding. The Shore hardness was measured as 60 A at room temperature using a durometer (LXD‐A, Sanryo Co., Ltd., Japan). The main materials and chemicals used in this study were as follows: metal mold substrate (304 stainless steel with the thickness of 0.3 mm), photomask (polyethylene terephthalate film with a thickness of 0.1 mm), substrate for performance testing (glass, Ra≈0.23 µm), photoresist (J‐1800 F‐705A, Jingbang Electronic Co., Ltd., China), developer (aqueous Na_2_CO_3_ solution, 10 wt.%), etchant (FeCl_3_ solution, 40°Bé), stripper (aqueous NaOH solution, 5 wt.%), cleaning solution (oxalic acid, 10 wt.%), anti‐adhesion coating agent (LW368, Longwei Cleanser Co., Ltd., China), and common solvents such as deionized water and ethanol.

### 4.2 Preparation of Bionic Adhesive Material

During the hot press process, a 100 µm thick silicone film was placed at the bottom of the mold to ensure the formation of mushroom‐shaped tips. This film was prepared by spin‐coating ∼ 5 g of RTV‐4130J (SILASTIC RTV‐4130‐J, Dow Chemical Company, USA) prepolymer on a glass plate at 3500 rpm for 60 s.

### 4.3 Fabrication of the Double‐Sided Mask

The double‐sided mask consists of two patterned PET (polyethylene terephthalate) film sheets bearing alignment marks along their edges. Under a microscope, the two films are precisely aligned using the edge alignment marks. Leveraging the thermoplastic property of the PET substrate, two opposite sides of the aligned films are permanently bonded by localized hot‐pressing. The remaining two sides are left open to allow insertion of the photoresist‐coated metal substrate. Details are shown in Figure S1.

### 4.4 Mold Release Coating on the Metal Mold

Prior to molding, the metal molds need to undergo an anti‐adhesion treatment: a robust anti‐adhesion fluoride coating was formed by five cycles of immersion in a fluoride solution (1 min) followed by oven‐drying (120°C, 5 min). The coating enhanced the release capability and durability of the mold.

### 4.5 Adhesive Performance Test

All test samples measured 1 × 1 cm^2^, with structures arranged in a hexagonal array at a fixed pitch of 600 µm. All adhesion tests were performed on clean glass (Ra≈0.23 µm). Samples (1 × 1 cm^2^) were tested on a Universal Mechanical Tester (Bruker, Germany) with a 0–200 N load sensor. A schematic of the experimental setup is provided in Figure S13. The testing involved applying a preload to sample, holding for 10 s, and then peeling at 0.5 mm s^−1^ until detachment. Each data point represents the mean of 3–5 measurements. Adhesion tests under low‐pressure environments were conducted using a custom‐modified fatigue tester (Figure S14), following the same peeling procedure. The load sensor had a range of 0–100 N.

### 4.6 Peeling Force Test

Peeling forces were measured using a Universal Mechanical Tester (Bruker, Germany). A sample (6 × 1 cm^2^) was fully adhered to a glass substrate on an angled support block. One end was connected to the force sensor, and a stable peeling angle was maintained by synchronizing the sensor's vertical motion (0.5 mm s^−1^) with the block's horizontal movement.

### 4.7 Vibration Performance Test

Adhesion under vibration was characterized using a compact precision vibration system (VE series, Hangzhou Yiheng Technology Co., Ltd.). The sample was fixed to the vibrating table, which operated continuously with real‐time monitoring via integrated acceleration and velocity sensors for closed‐loop control (Figure S15).

### 4.8 Statistical Analysis

All quantitative data in this study are presented as mean ± standard deviation. Statistical analysis was performed using Origin.

## Author Contributions

**Keju Ji**: conceptualization, funding acquisition, resources, project administration, supervision, writing – review and editing, methodology, validation. **Xinran Xu**: data curation, formal analysis, investigation, validation, methodology, visualization, writing – original draft. **Jian Chen**: methodology, data curation, visualization. **Wenjie Chen**: visualization, data curation, software. **Xiaolei Zhu**: validation, visualization, software. **Haixiang Ma**: formal analysis, visualization. **Xingyu Jiang**: formal analysis, visualization. **Changhong Linghu**: methodology, supervision, writing – review and editing, project administration, funding acquisition, conceptualization, investigation. **Zhendong Dai**: methodology, supervision, investigation.

## Consent

This study involved human participants. The experimental activity (Participants raised heels to achieve a controlled suspension.) was assessed as minimal risk. Prior to the experiment, all participants were fully informed of the procedural details and voluntarily provided written informed consent. No adverse events occurred during the study.

## Conflicts of Interest

The authors declare no conflicts of interest.

## Supporting information




**Supporting File**: advs77709‐sup‐0001‐SuppMat.docx.

## Data Availability

The data that support the findings of this study are available from the corresponding author upon reasonable request.
