# Supplementary material for: A Scalable Double‐Sided Wet Etching Strategy for Large‐Area Mushroom‐Shaped Bionic Adhesives
Source: Adv Sci (Weinh). 2026 Sep 11:e77709. Online ahead of print. doi: 10.1002/advs.77709 (PMC13564180; doi:10.1002/advs.77709)
Supplement: Supplementary file 1 [file ADVS-9999-e77709-s001.docx]

Supporting Information

**A Scalable Double-Sided Wet Etching Strategy for Large-Area Mushroom-Shaped Bionic Adhesives**

Keju Ji^*^, Xinran Xu, Jian Chen, Wenjie Chen, Xiaolei Zhu, Haixiang Ma, Xingyu Jiang, Changhong Linghu^*^, Zhendong Dai

Keju Ji, Xinran Xu, Jian Chen, Wenjie Chen, Xiaolei Zhu, Haixiang Ma, Xingyu Jiang, Zhendong Dai

Jiangsu Key Laboratory of Bionic Materials and Equipment, College of Mechanical and Electrical Engineering, Nanjing University of Aeronautics and Astronautics, Nanjing 210016, China

Changhong Linghu

Department of Mechanical Engineering, College of Engineering, City University of Hong Kong, Hong Kong, 999077, China

**Figure S1. Fabrication of the double-sided photomask.** (a) Print two PET films with alignment marks. (b) Alignment under a microscope and bond via localized hot-pressing. (c) Insertion of the metal substrate.

During demolding, the adhesive structure is pulled from the mushroom-shaped mold cavities. As depicted in Figure S2, under the combined action of this pulling force and the constraint from the mold cavity wall, the neck region of the structure undergoes reversible elastic deformation. Demolding is attributed to the following synergistic mechanisms: (1) The fluorinated layer on the mold surface creates a low‑surface‑energy interface, which significantly reduces the peel resistance required for release. (2) The selected solid silicone rubber possesses a high fracture elongation (571.39%) and a low Young’s modulus (8.17 MPa). This allows the structure to accommodate large local strains during demolding without tearing and, upon stress release, to recover elastically and accurately replicate the original designed geometry.

**Figure S2. Schematic of a mushroom‑shaped structure during the demolding process.**

**Optimization of Etching Parameters**

**(1) Selection of etchant concentration:**

The etching of stainless steel with ferric chloride (FeCl₃) is typically performed at concentrations between 30°Bé and 50°Bé^[1,2]^. To identify the optimum concentration, immersion tests were carried out at 25 °C. Four identical stainless‑steel sheets (5 cm × 5 cm × 0.3 mm) were etched for 2 h in FeCl₃ solutions of 20°Bé, 30°Bé, 40°Bé, and 50°Bé. The measured mass losses were 0.789 g, 3.038 g, 3.278 g, and 1.44 g, respectively, revealing a non-monotonic trend where the etching rate increases to a maximum before declining (Figure S3a).

Lower concentrations markedly reduce the etching rate, thereby enhancing process controllability while prolonging the etching duration. Higher concentrations, owing to the high abundance of Fe³⁺ ions, offer strong oxidizing power and thus a high etching rate. Nevertheless, the associated increase in viscosity hinders mass transfer, leading to the accumulation of reaction by-products. This accumulation forms a concentration polarization layer at the interface, which can inhibit the etching reaction and reduce its effective rate^[2]^. Additionally, in masked etching, the inherent isotropy of the chemical process is amplified at high concentrations, aggravating undercut and compromising feature definition.Based on these results, a concentration of 40°Bé was selected for this study, as it offers a suitable balance between etching rate and undercut control.

**(2) Selection of Etching Temperature**

Reported etching temperatures for FeCl₃ typically range from 40 to 55 °C^[3–5]^. Elevated temperature provides greater activation energy for Fe³⁺ ions, thereby increasing the etching rate. However, higher temperature also accelerates the hydrolysis of FeCl₃, which generates insoluble iron hydroxide precipitates. This side reaction consumes Fe³⁺, lowers the solution acidity, shortens the etchant’s usable life, and can lead to particulate clogging within etched cavities.

To determine the optimum temperature, etching trials were conducted on patterned plates (450 μm mask opening) at 20, 30, 40, and 50 °C. Etch depth and undercut were measured with a laser confocal microscope (LEXT OSL5000, Olympus, Japan), and the etch factor (EF) was derived. As shown in Figure S3b, etching at 40 °C resulted in a depth of 26.99 μm and an EF of 0.647, reflecting favorable uniformity and controllability. In contrast, although 50 °C produced the greatest depth (37.31 μm), the corresponding EF was lower (0.594), indicating enhanced lateral etching, increased edge roughness, and diminished surface quality.

Consequently, 40 °C was selected as the operating temperature. This condition offers a balanced compromise: it maintains efficient etching while limiting the formation of deleterious by‑products.

**Figure S3. Etching parameter selection**. (a) Weight loss at different etchant concentrations. (b) Etch depth and etch factor at different temperatures.

**Roughness evolution during etching and the impact on mold release**

Under constant etching conditions, a positive correlation exists between the etching rate and the surface roughness of the resulting cavities throughout the kinetic process. In the FeCl₃ solution etching system for 304 stainless steels, a higher etching rate intensifies reaction heterogeneity. This readily induces selective etching, thereby increasing the surface roughness (*Ra*). To quantify this relationship, the cavity bottom roughness with etching time was systematically measured to track evolution. The measurements were conducted at a fixed mask opening size of 250 μm with a time interval of 5 min (Figure S4). The results show a non-monotonic trend in surface roughness, which increases rapidly initially and then decreases slowly over time. This trend aligns completely with that of the etching rate, confirming their positive correlation throughout the kinetic process, which is consistent with most reported studies^[6,7]^.

**Figure S4. Surface roughness of the etched cavities at different etching times.**

Surface roughness can affect demolding mainly through mechanical interlocking and increased interfacial friction. However, the surface roughness obtained in the molds does not hinder the release of the adhesive material. The main reasons are as follows. (1) The solid silicone rubber used exhibits high elongation (>570%) and a low Young's modulus (~8.17 MPa). Its high flexibility and elasticity allow materials to accommodate surface microscopic via localized large deformation during demolding, effectively relieving stress concentration and preventing tearing. (2) An anti-adhesion agent was applied to the metal mold before demolding, which further reduced interfacial friction. (3) The optimized etching parameters used in this study (*T_SE_* = 40 min, *T_DE_* = 11 min) correspond to a relatively low-roughness stage of the etching process. This parameter selection was constrained by the etching failure time and the required morphology of the mushroom-shaped structure. Therefore, the process avoided the unstable etching stage near the roughness peak and minimizing its potential influence of surface roughness on demolding from the outset.

As shown in Figure S5 with the etching process for a mask size of 300 μm as an example, etching failure occurs when the processing time reaches 50 minutes. In contrast, samples etched for 45 minutes show no adhesion between adjacent cavities, indicating a stable etching state before failure.

**Figure S5. Both 2D and 3D views of a single metal cavity captured at five-minute intervals during single-sided etching with a 300 μm mask.**

After removing the cross-linked ink, the ink morphology exhibited a reduced mask layer size. As shown in Figure S6, when the mask diameter was 550 μm, the diameter of exposed etchable area after ink removal measured 515 ± 3.2 μm, with a structural spacing of 600 micrometers. Consequently, the maximum side etching experienced was 40 μm, which is less than the side etching achievable within the first five minutes of etching.

**Figure S6. Contour curve of the ink layer after exposure.**

**Effect of Preload and Retraction Speed on Adhesive Strength**

For the preload effect, the adhesion force increases with increasing preload and then tends to plateau. At the preload range of 0 to 10 N, the mushroom-shaped microstructures only partially contact the substrate. Increasing the preload promotes elastic deformation of the microstructures and improves conformal contact with the substrate. This increases the real contact area and therefore enhances the adhesion force. When the preload becomes sufficiently high, most microstructures are already in effective contact with the substrate. The real contact area then approaches saturation, and the increase in adhesion force becomes less pronounced.

For fibrillar adhesive structures with a high aspect ratio, excessive preload may induce buckling or collapse of the microstructures. This can reduce the effective contact area and lead to a decrease in adhesion. However, the aspect ratio of the structures used in this study is lower than 1.5. Therefore, buckling was unlikely to occur within the tested preload range. As a result, Figure S7 mainly shows the increase and subsequent plateau of adhesion with increasing preload.

For the retraction-speed effect, the adhesion force increases with increasing retraction speed. This behavior is typical for soft viscoelastic adhesive materials^[8]^. During rapid detachment, the silicone rubber undergoes faster deformation. The molecular chains have less time to relax, which leads to higher internal friction and greater energy dissipation. As a result, a larger peak separation force is required. This rate-dependent adhesion behavior is consistent with classical studies on viscoelastic adhesion and fracture of soft materials^[9]^.

**Figure S7. Adhesion performance tests of samples.** (a) Adhesion strength as a function of preload for six samples. A retraction speed of 0.5 mm s^-1^ and a holding time of 10s were applied in tests. (b) Adhesion strength as a function of retraction speed for six sample groups. A preload of 13 N and a holding time of 10s were applied in tests.

**Figure S8. Normal adhesion performance of a flat pillar structure under atmospheric conditions.** The retraction speed was 0.5 mm s^-1^.

**Details of the Finite Element Simulations**

In this work, the geometric model (a two-dimensional axisymmetric model) in finite element simulation mainly consists of an adhesive structure and a backing layer. The thickness of the backing layer is 0.2mm, and the rest of the structure dimensions are the same as the actual dimensions.

During the simulation, we introduced a traction-separation law to describe the cohesive contact Property (as shown in Figure S9). The formula of the cohesive constitutive law is:

$$F=\left\{ \begin{aligned} F_{n} \\ F_{s} \\ F_{t} \end{aligned} \right\}=A\left[ \begin{aligned} K_{nn} K_{ns} K_{nt} \\ K_{ns} K_{ss} K_{st} \\ K_{nt} K_{st} K_{tt} \end{aligned} \right]\left\{ \begin{aligned} \delta_{n} \\ \delta_{s} \\ \delta_{t} \end{aligned} \right\}=AK\delta$$

where *F*_n_ represents the normal separation force acting at the interface during detachment, while *F_s_* and Ft represent the two shear separation forces. The corresponding separation displacements in the three directions are represented by *δ_n_*, *δ_s_*, and *δ_t_*, respectively; A represents to the total interfacial area, and *K* represents the interfacial stiffness matrix. The initial damage criterion for the interface is defined by the maximum nominal stress criterion, and its constitutive relation is given as follows:

$$Max\left\{ \frac{\sigma_{n}}{\sigma_{Nmax}},\frac{\delta_{s}}{\delta_{smax}} ,\frac{\delta_{t}}{\delta_{tmax}} \right\}=1$$

**Figure S9. Sketch of the traction-separation response for cohesive behavior of adhesive structures in the numerical simulations.**

In the finite element simulation of the separation process, the substrate base was fully fixed. The simulation replicated the actual testing procedure, which involved applying a preload followed by uplift. Thus, the adhesion process was divided into two stages: a preload of 20 mN was applied in the first stage, and a displacement constraint of 0.09 mm was imposed in the second stage. A reference point was defined at the center of the upper surface of the structural backing layer to extract the separation force–displacement curve. Within the ABAQUS/Standard contact analysis framework, the small-sliding contact was adopted to define the contact interaction, accounting for minor relative sliding or rotation between the contacting surfaces. The criterion of interface fracture damage is the maximum nominal stress; the specific parameters are 0.15, 0.15, 0.15, the interface stiffness is 50 N mm^-1^, and the fracture energy is 1E-9 mJ. In order to improve the convergence of the model, the viscosity coefficient of the interface is set to 1E-15, the triangle diagram for cohesive mode is as above.

**Figure S10. Finite element simulation of adhesion behavior for adhesive structures with different taper angles of the expanded tip (****𝜃).** (a, b) Dynamic deformation processes of structures with low and high taper angles; contour plots represent internal stress distributions. (c) Contact stress profiles at the structure to substrate interface. (d) Variation of adhesion force with simulation time for different structures. (e) Variation of adhesion energy with simulation time for different structures.

**Figure S11. Adhesion performance of bionic adhesive materials on convex surfaces under a vacuum environment.** (a, c) Adhesion strength as a function of vacuum level for three samples (*D_m_*-500 and Flat pillar) at a fixed retraction speed (v=0.5 mm s^-1^). (b, d) Adhesion strength as a function of retraction speed for three samples (*D_m_*-500 and Flat pillar) at a fixed vacuum level of 0.02 MPa.

**Figure S12. Mechanical characterization of tear-resistant silicone.** (a) Hardness test. (b) Stress-strain curve in uniaxial tensile test, showing Young's modulus measurement of 4.86 MPa, and elongation at break of 571.39%. (c) The material exhibits excellent deformability; images show its state before and after stretching.

**Figure S13. Schematic of the mechanical testing system for adhesive materials under atmospheric conditions.** (a) Diagram of the UMT platform (Universal Mechanical Tester, Bruker, Germany) for adhesion measurement, which includes a high-precision force transducer (range: 0-200 N) and XYZ 3D motion control modules. (b) A representative adhesion force–displacement curve.

This adheison test system under vacuum is shown in Figure S14. The core component is a pressure-resistant sealed chamber. A manometer (I) monitors the chamber pressure, which can be reduced to 0.02 MPa to simulate low-pressure environments from near-Earth orbit to deep space (Fig. 3a). The preload module (III) applies a controlled pressure via a precision displacement stage, ensuring full contact between the test sample (V) and the glass substrate (VI). The force sensor (IV) records the force-displacement curve during vertical peeling (II).

**Figure S14. Schematic of the mechanical testing system for adhesive materials under vacuum.**

A precision vibration system (VE-5120, EHS) was used with the glass substrate rigidly mounted on the vibration table for the adheison test under vibration, as shown in Figure S15. The bionic adhesive sample was installed perpendicular to the substrate. Control sensors on the load side enabled real-time monitoring. Vibration conditions (frequency and amplitude) are precisely set by the controller.

**Figure S15. Schematic of the vibration test setup.**

Table S1. Comparison of area of adhesive materials, adhesion strength, retention rate under vacuum, vibration resistance, durability between this work and previously reported literature.

| Ref. | Materials | Fabrication methods | Materials of mold | Area of adhesive materials | Adhesive  micro-structure | Normal Adhesive properties | Retention rate under vacuum | Vibration resistance | Durability |
| --- | --- | --- | --- | --- | --- | --- | --- | --- | --- |
| 10 | Sylgard 184 | Diamond Tool Cutting | Aluminum | N/A | Wedge-structured | ~10.5kPa (3mm min^-1^) | Hardly decay | N/A | N/A |
| 11 | TPU and silicone rubber | Electrically responsive self-growing technique | Direct Fabrication | 7cm×7cm | Mushroom-shaped with rigid core and soft shell | ~96.67kPa (1mm min^-1^) | N/A | N/A | Yes |
| 12 | UV-light-curable polyurethane acrylates | Roll-to-roll scaled-up manufacturing | Silicon, Silicone rubber | N/A | Mushroom-shaped | ~130kPa (0.6mm min^-1^) | N/A | N/A | Yes |
| 13 | PDMS and polyurethane foam | Double-sided exposure process with physical cuts | Photoresist | 2cm×2cm | Mushroom-shaped with mechanically isolated units | ~24.5N  (5mm min^-1^) | N/A | Yes | N/A |
| 14 | RTV-4130-J | Microstructured electroformed mold  and roll-to-roll scaled-up manufacturing | Nickel | N/A | Mushroom-shaped tips | ~95 KPa  (30mm min^-1^) | N/A | N/A | N/A |
| 15 | Ecoflex 00–30 | Moulding by casting | Photopolymer Resin | 4cm×4cm | Hierarchical structure | ~20.7N  (30mm min^-1^) | N/A | Yes | Yes |
| 16 | Fluoroelastomer | Laser-structured mold-assisted hot embossing | Aluminum | 12cm^2^ | Cylinder without tips | ~106.3 KPa  (30mm min^-1^) | 17.3% | Yes | Yes |
| 17 | Sylgard 184 | Photoresist mold-casting | Photoresist | 170cm^2^ | Mushroom-shaped tips | ~118 KPa  (30mm min^-1^) | 32.6% | N/A | Yes |
| This work | Silicone rubber | Wet-etched mold and hot embossing | Stainless Steel | 30cm×30cm | Mushroom-shaped tips | ~170kPa (30mm min^-1^)  ~52.1kPa (6mm min^-1^) | 1.71% | Yes | Yes |

Supplemental references

[1] O. Çakır, “Effects of Etchant Concentration and Process Temperature in Chemical Milling of 430 Stainless Steel with FeCl3,” *Materials Today: Proceedings* (2023): S221478532304035X, https://doi.org/10.1016/j.matpr.2023.07.165.

[2] O. Çakır, “Study of Etch Rate and Surface Roughness in Chemical Etching of Stainless Steel,” *Key Engineering Materials* *364–366* (2007): 837–842, https://doi.org/10.4028/www.scientific.net/KEM.364-366.837.

[3] T. Tiwari, A. Dvivedi, and P. Kumar, “Investigations on the Fabrication of a Patterned Tool by Chemical Etching,” *Materials and Manufacturing Processes* *36*, no. 16 (2021): 1840–1852, https://doi.org/10.1080/10426914.2021.1926491.

[4] F. Xin, T. Ma, Y. Chen, et al., “Study on Chemical Spray Etching of Stainless Steel for Printed Circuit Heat Exchanger Channels,” *Nuclear Engineering and Design* *341* (2019): 91–99, https://doi.org/10.1016/j.nucengdes.2018.10.022.

[5] P. Nageswara Rao, and D. Kunzru, “Fabrication of Microchannels on Stainless Steel by Wet Chemical Etching,” *Journal of Micromechanics and Microengineering* *17*, no. 12 (2007): N99–N106, https://doi.org/10.1088/0960-1317/17/12/N01.

[6] D. Agrawal, and D. Kamble, “Optimization of Photochemical Machining Process Parameters for Manufacturing Microfluidic Channel,” *Materials and Manufacturing Processes* *34*, no. 1 (2019): 1–7, https://doi.org/10.1080/10426914.2018.1512115.

[7] H.-C. Jeong, D.-W. Kim, G.-M. Choi, et al., “The Effect of Spray Characteristics on the Etching of Invar Alloy with FeCl3 Solution,” *International Journal of Precision Engineering and Manufacturing* *10*, no. 4 (2009): 107–114, https://doi.org/10.1007/s12541-009-0078-0.

[8] A. Ghatak, L. Mahadevan, J. Y. Chung, et al., “Peeling from a Biomimetically Patterned Thin Elastic Film,” *Proceedings of the Royal Society of London. Series A: Mathematical, Physical and Engineering Sciences* *460*, no. 2049 (2004): 2725–2735, https://doi.org/10.1098/rspa.2004.1313.

[9] C.-Y. Hui, J. M. Baney, and E. J. Kramer, “Contact Mechanics and Adhesion of Viscoelastic Spheres,” *Langmuir* *14*, no. 22 (1998): 6570–6578, https://doi.org/10.1021/la980273w.

[10] D. Tao, X. Gao, H. Lu, et al., “Controllable Anisotropic Dry Adhesion in Vacuum: Gecko Inspired Wedged Surface Fabricated with Ultraprecision Diamond Cutting,” *Advanced Functional Materials* *27*, no. 22 (2017): 1606576, https://doi.org/10.1002/adfm.201606576.

[11]	H. Tian, D. Wang, Y. Zhang, et al., “Core–Shell Dry Adhesives for Rough Surfaces via Electrically Responsive Self-Growing Strategy,” *Nature Communications* *13*, no. 1 (2022): 7659, https://doi.org/10.1038/s41467-022-35436-6.

[12]	J. Kim, N. Krishna‐Subbaiah, Y. Wu, et al., “Enhanced Flexible Mold Lifetime for Roll‐to‐Roll Scaled‐Up Manufacturing of Adhesive Complex Microstructures,” *Advanced Materials* *35*, no. 2 (2023): 2207257, https://doi.org/10.1002/adma.202207257.

[13] D. Wang, H. Tian, H. Liu, et al., “Bioinspired Dry Adhesives for Highly Adaptable and Stable Manipulating Irregular Objects under Vibration,” *Advanced Science* *10*, no. 21 (2023): 2302512, https://doi.org/10.1002/advs.202302512.

[14]	Q. Li, K. Ji, J. Zhao, et al., “Manufacturing of Bionic Adhesion Microstructure with Expanded Ends Based on Electroplating in the Restricted Area,” *ACS Applied Materials & Interfaces* *16*, no. 37 (2024): 49985–49992, https://doi.org/10.1021/acsami.4c11410.

[15] J. Kim, G. W. Hwang, M. Song, et al., “A Reversible, Versatile Skin‐Attached Haptic Interface Platform with Bioinspired Interconnection Architectures Capable of Resisting Sweat and Vibration,” *Advanced Functional Materials* *34*, no. 17 (2024): 2311167, https://doi.org/10.1002/adfm.202311167.

[16]	J. Zhang, T. Huo, Y. Ji, et al., “Biomimetic Manipulation of Smooth Solid Surfaces for Vacuum High-Temperature and Vibration Environments,” *Journal of Bionic Engineering* *22* (2025): 755–766, https://doi.org/10.1007/s42235-024-00645-w.

[17]	C. Li, J. Zhao, J. Chen, et al., “Effect of Thermal Reflow on Microstructural Morphology and Contact Mechanics in the Photo‐Lithographic Fabrication of Biomimetic Adhesive Materials,” *Small Methods 9, no 8* (2025): 2402123, https://doi.org/10.1002/smtd.202402123.
